# Protective Activity of *Streptococcus pneumoniae* Spr1875 Protein Fragments Identified Using a Phage Displayed Genomic Library

**DOI:** 10.1371/journal.pone.0036588

**Published:** 2012-05-03

**Authors:** Angela Cardaci, Salvatore Papasergi, Angelina Midiri, Giuseppe Mancuso, Maria Domina, Veronica Lanza Cariccio, Francesca Mandanici, Roberta Galbo, Carla Lo Passo, Ida Pernice, Paolo Donato, Susanna Ricci, Carmelo Biondo, Giuseppe Teti, Franco Felici, Concetta Beninati

**Affiliations:** 1 The Elie Metchnikoff Department, Università di Messina, Messina, Italy; 2 Dipartimento di Scienze della Vita M. Malpighi, Università di Messina, Messina, Italy; 3 Novartis Vaccines and Diagnostics, Messina, Italy; 4 Dipartimento di Biotecnologia, Università di Siena, Siena, Italy; 5 Dipartimento S.T.A.T., Università del Molise, Pesche (IS), Italy; The Scripps Research Institute, United States of America

## Abstract

There is considerable interest in pneumococcal protein antigens capable of inducing serotype-independent immunoprotection and of improving, thereby, existing vaccines. We report here on the immunogenic properties of a novel surface antigen encoded by ORF *spr1875* in the R6 strain genome. An antigenic fragment encoded by *spr1875*, designated R4, was identified using a *Streptococcus pneumoniae* phage displayed genomic library after selection with a human convalescent serum. Immunofluorescence analysis with anti-R4 antisera showed that Spr1875 was expressed on the surface of strains belonging to different serotypes. Moreover, the gene was present with little sequence variability in 27 different pneumococcal strains isolated worldwide. A mutant lacking Spr1875 was considerably less virulent than the wild type D39 strain in an intravenous mouse model of infection. Moreover, immunization with the R4 recombinant fragment, but not with the whole Spr1875 protein, induced significant protection against sepsis in mice. Lack of protection after immunization with the whole protein was related to the presence of immunodominant, non-protective epitopes located outside of the R4 fragment. In conclusion, our data indicate that Spr1875 has a role in pneumococcal virulence and is immunogenic. As the R4 fragment conferred immunoprotection from experimental sepsis, selected antigenic fragments of Spr1875 may be useful for the development of a pneumococcal protein-based vaccine.

## Introduction

*Streptococcus pneumoniae*, or pneumococcus, is an extracellular human respiratory pathogen causing sinusitis, otitis media, pneumonia, sepsis and meningitis. Invasive pneumococcal infections are an important cause of mortality and morbidity worldwide, especially among young children and the elderly [1]. Pneumococci cause at least 1 million deaths worldwide every year, mostly as a result of community-acquired pneumonia [2]. Moreover, antibiotic resistance is increasing at an alarming rate among pneumococcal clinical isolates. The 23-valent polysaccharide vaccine provides partial, serotype-specific protection in adults, but has limited efficacy in young children. Although conjugate vaccines are effective also in children, protection is limited to the serotypes present in vaccine formulation and serotype replacement is threatening to decrease vaccine efficiency. This is exemplified by the increase, in the 7-valent vaccine era, of invasive disease caused by strains of serotype 19A, a serotype not included in this vaccine [3], [4]. Therefore, the development of serotype-independent vaccines targeting protein virulence factors is being actively pursued and several pneumococcal proteins have been proposed as potential vaccine candidates [5].

We have recently used a lambda phage displayed whole genome library to identify several antigenic pneumococcal fragments, based on their ability to bind to serum antibodies from patients convalescing from pneumococcal infection or from experimentally infected mice. This powerful approach allowed the identification of a large panel of B-cell epitopes within known virulence factors or protective antigens, including members of the choline-binding, histidine-triad and zinc metalloproteinase families [6]. Furthermore, we identified new antigenic regions matching the sequence of a novel pneumococcal adhesin, which was designated as plasminogen and fibronectin binding protein B [7]. We report here on the identification of a novel fragment designated as R4, which is encoded by ORF *spr1875* in the R6 genome. The *spr1875* gene was found to be conserved amongst pneumococcal strains isolated from different geographical areas. In an experimental model of sepsis, a mutant strain devoid of Spr1875 was attenuated in virulence. Moreover immunization with the R4 fragment of Spr1875 conferred protection from intravenous challenge with virulent pneumococci.

## Results

### Spr1875 is expressed on the bacterial surface

**Figure 1:**
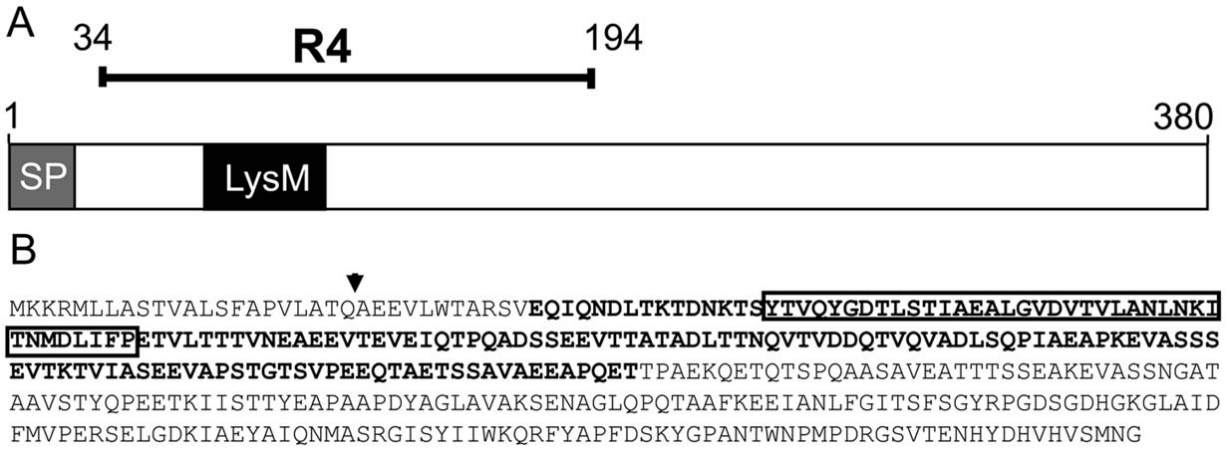
Schematic structure and deduced amino acid sequence of protein Spr1875 of *Streptococcus pneumoniae*. (A) SP, signal peptide; LysM, LysM peptidoglycan interaction domain. The extension of the fragment isolated from the phage display library (R4) is also indicated. (B) Amino acid sequence of Spr1875, as deduced from ORF *spr1875* in the strain R6 genome. The arrow indicates the predicted signal peptidase cleavage site, according to SignalP v3.0 predictions. The LysM domain and the R4 fragment are indicated by the box and by the bold characters, respectively.

A lambda phage displayed library of the pneumococcal genome (strain R6) was previously used to identify several antigenic fragments based on their reactivity with human serum antibodies [6]. By this approach, in the present study, we identified a novel 161 amino acid-long fragment, herein referred to as R4, using serum antibodies from a patient convalescing from invasive pneumococcal disease. The sequence matched ORF *spr1875* of the *S. pneumoniae* R6 strain genome (GenBank accession no. AE007317), encoding a 380 amino acid-long protein with an N-terminal peptidoglycan interaction lysine motif (LysM) domain, which is found in cell wall degrading enzymes and in virulence factors (Fig. 1). The predicted protein sequence of Spr1875 contains a leader peptide with a leader sequence and a cleavage site present in variety of streptococcal surface proteins. We next produced a recombinant R4-GST fusion protein and assessed its ability to bind to serum antibodies from patients recovering from pneumococcal infection. It was found that a high proportion of such serum samples, but not control samples, displayed high anti-R4 antibody titers (Table S1).

**Figure 2:**
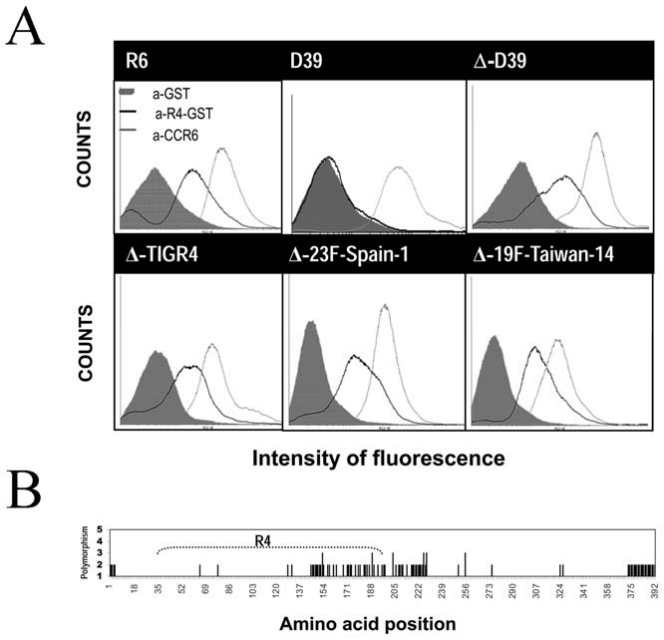
Surface expression of Spr1875 and *spr1875* gene polymorphism in different pneumococcal strains. (A) Immunofluorescence flow cytometry analysis of different *S. pneumoniae* strains using anti-R4-GST mouse serum (black lines). Anti-GST (grey peaks) and anti-CCR6 (grey lines) murine sera were used as negative and positive controls, respectively. Δ-D39, Δ-TIGR4, Δ-23F-Spain-1 and Δ-19F-Taiwan-14 are unencapsulated mutants of D39, TIGR4, 23F-Spain-1 and 19F-Taiwan-14, respectively. (B). Schematic representation of *spr1875* deduced amino acid sequence variability within *S. pneumoniae*. Twenty-seven strains were used for gene variability analysis. Abscissa, amino acid position; ordinate, degree of polymorphism. The baseline represents the sequence of the P1031 *S. pneumoniae* strain, which was the longest sequence. The height of the line indicates the total number of different amino acids found at that particular position.

To assess whether the Spr1875 protein is actually expressed on the bacterial surface, we used R4-GST to immunize mice. Mice were also immunized with recombinant GST and CCR6, a crude pneumococcal surface protein extract, to obtain negative and positive control sera, respectively. Figure 2A (upper panels) shows that sera from mice immunized with R4 fused to GST, but not sera from mice immunized with GST alone, bound to the surface of the rough R6 strain, or to an unencapsulated D39 mutant (Δ-D39, Fig. 2A). Antibodies from CCR6-immunized mice positively reacted with all strains tested, as expected. In addition, anti-R4 antibodies did not bind to the surface of the parental D39 encapsulated strain (Fig. 2A). These data indicate that Spr1875 is expressed on the bacterial surface, but is largely masked by the polysaccharide capsule.

### Spr1875 is expressed in *S. pneumoniae* strains of different serotypes

Next, we investigated whether Spr1875 is expressed in strains of different pneumococcal serotypes. To this end, the unencapsulated derivatives of TIGR4, 23F-Spain-1, and 19F-Taiwan-14 were examined by immunofluorescence flow cytometry analysis using anti-R4 mouse sera (Fig. 2A, lower panels). Anti-R4 antibodies bound to the surface of each of these strains, indicating that immunoreactive Spr1875 is expressed on pneumococcal strains of different serotypes and geographical distribution. Next, we compared the *spr1875* gene sequences in 27 pneumococcal strains using sequences available in DNA data bases. Figure 2B shows the degree of sequence variability at different positions in the deduced amino acid sequences. These sequences showed a remarkable degree of conservation with no or only few variations at each amino acid position. Such variations are mostly located in the central portion and at the C- and N-terminal regions of the protein sequence. A complete list of the aligned deduced amino acid sequences is reported in Table S2 in supplemental material, which shows that the 27 sequences belonged to one of 14 different sequence types.

### Spr1875 is required for in vivo pneumococcal growth

**Figure 3:**
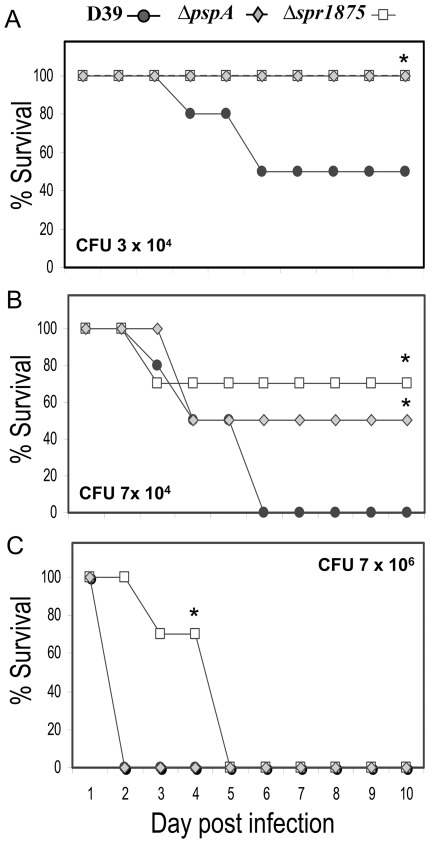
Lethality induced in mice by a D39 mutant (Δ*spr1875*) lacking Spr1875. Wild type D39 and a mutant lacking PspA (Δ*pspA*) were used as controls. CD1 mice were challenged intravenously with 3×10^4^ (A), 7×10^4^ (B) or 7×10^6^ (C) CFU. Results are from one experiment per challenge dose involving 10 mice per group. *, statistically different from D39 strain, as assessed by Kaplan-Meier estimator of survival.

The pathogenicity of pneumococci has been attributed to various virulence factors, mostly located on its surface. To evaluate whether Spr1875 has an impact on pneumococcal virulence, we constructed Δ*spr1875*, a deletion mutant of the virulent, encapsulated D39 strain. Spr1875 was not detectable, by western blot, in *Δspr1875* cell lysate using anti-R4 mouse serum (Fig. S1). As a control, we also constructed Δ*pspA*, an isogenic D39 mutant devoid of PspA, a well-characterized virulence factor that prevents complement deposition on the bacterial surface [8]. Figure 3 shows survival plots of mice inoculated intravenously with the wild-type D39 strain and Δ*spr1875* or Δ*pspA* mutants. Using the lower bacterial doses (Fig. 3A and 3B), we observed markedly increased survival in both Δ*spr1875*- and Δ*pspA*-challenged mice, as compared with the wild type D39 strain. The highest challenge dose (7×10^6^ CFU) killed all mice within 5 days in each of the D39-, Δ*spr1875*- or Δ*pspA*-infected groups (Fig. 3C). However, with this dose, Δ*spr1875*-infected mice had a significantly increased survival time relative to either D39- or Δ*pspA*-infected mice. In each experiment, pneumococci were confirmed as the cause of death by organ colony counts in moribund animals. These data show that the *spr1875*-deficient strain is attenuated in virulence, suggesting that the Spr1875 protein may play a role in pneumococcal sepsis.

### Protective activity of the R4 peptide fragment

**Figure 4:**
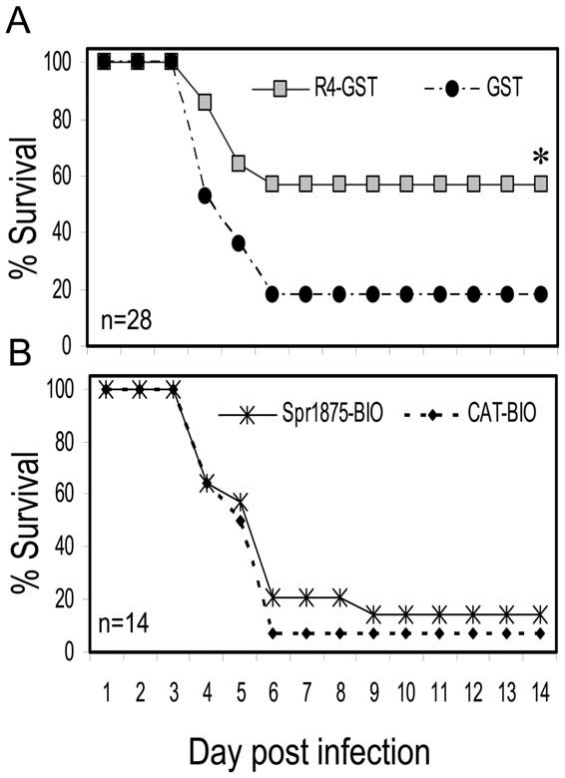
Protection induced by immunization with recombinant R4-GST or by the whole recombinant Spr1875 protein fused to a biotinylated tag (Spr1875-BIO). (A) Groups of CD1 mice were immunized with R4-GST or with GST, used as a negative control, and challenged with the D39 strain (1×10^5^ CFU). Results represent cumulative data from 3 experiments; n = 28, total number of animal per each group. (B) Groups of CD1 mice were immunized with Spr1875-BIO or with CAT-BIO, used as a negative control, and challenged with the D39 strain (1×10^5^ CFU). Results represent data from one experiment involving 14 animals per group. *, statistically different (p<0.05) from mice challenged with D39 strain, as assessed by Kaplan-Meier estimator of survival.

To assess whether immunization with the recombinant R4 fragment had protection-eliciting activity against pneumococcal infection, groups of mice were immunized with 50 µg of the R4-GST fusion protein. After three administrations, all mice had R4-specific serum antibody titers ranging from 1 8,000 to 1 64,000 (data not shown). R4-GST-immunized mice were challenged with an approximately 90% lethal dose (1×10^5^ CFU) of the D39 strain. Lethality was observed for 14 days and compared with that observed in mice immunized with the GST tag only. Cumulative data from three experiments indicated that immunization with the R4-GST fragment resulted in 57% (16 mice out of 28) survival, while only 18% (5 mice out of 28) of the GST-immunized animals survived (p<0.05; Fig. 4A). Further data indicated that the protection induced by immunization with R4-GST was antibody-mediated, since it could be transferred to unimmunized animals by pooled sera from animals immunized with R4-GST, but not from those immunized with GST alone (Fig. S2).

**Figure 5:**
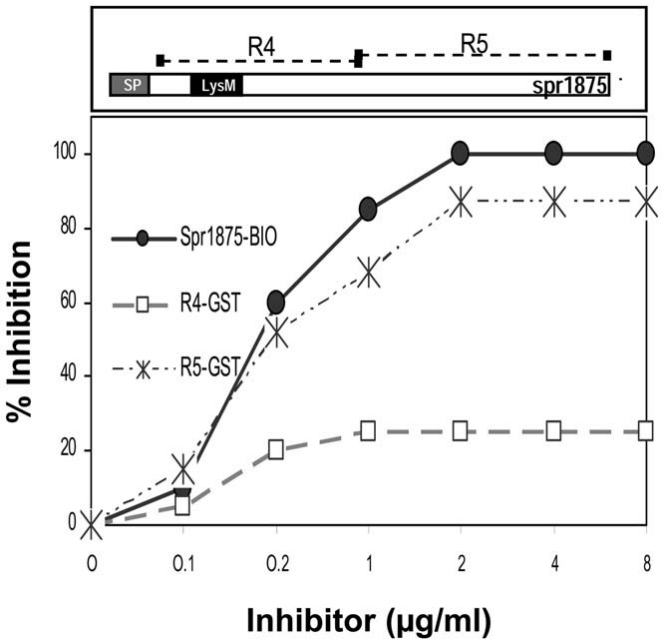
The antibody response to recombinant Spr1875 is predominantly directed against epitopes located outside of the R4 fragment. Shown are inhibition ELISA experiments measuring the reactivity against Spr1875-GST of a serum pool (with an antibody titer of 1 16,000) from animals immunized with anti-Spr1875-BIO in the presence of inhibitors. The recombinant Spr1875 fragments R4-GST and R5-GST (see upper diagram) were used as inhibitors. Results are from one experiment representative of three.

We next recombinantly produced the whole Spr1875 protein fused to a biotin (Spr1875-BIO) or to a GST (Spr1875-GST) tag and used them for immunizing animals as in the experiments described above. Surprisingly, neither Spr1875-BIO nor Spr1875-GST immunization resulted in significant protection when compared with immunization with the respective negative control protein (Fig. 4B and Fig. S3). Based on these data we hypothesized the induction of a different antibody response to the whole Spr1875 protein as compared to immunization with the R4 fragment. To gain insights into the portion of the Spr1875 protein against which serum antibodies were directed, we expressed a recombinant fragment, designated R5, fused to GST (R5-GST). This fragment encompassed the whole length of the Spr1875 protein excluding the R4 fragment (Fig. 5, upper panel). Next, we conducted inhibition experiments in which R4-GST and R5-GST were used to inhibit the reactivity of anti-Spr1875 serum antibodies from Spr1875-BIO-immunized animals. In these ELISA experiments, Spr1875-GST was used as a coating antigen and antibody titers were measured in the presence and in the absence of inhibitors. Figure 5 shows that up to 87% of the reactivity of such sera was inhibited by R5-GST, while only 25% was inhibited at saturation by R4-GST. The latter fragment, however, completely inhibited reactivity of anti-Spr1875 antibodies in sera from R4-GST-immunized animals (data not shown). These data indicate that the large majority of serum antibodies from animals immunized with the whole Spr1875 protein was directed against the R5, and not the R4, portion of the molecule.

### Protection induced by other Spr1875 fragments

**Figure 6:**
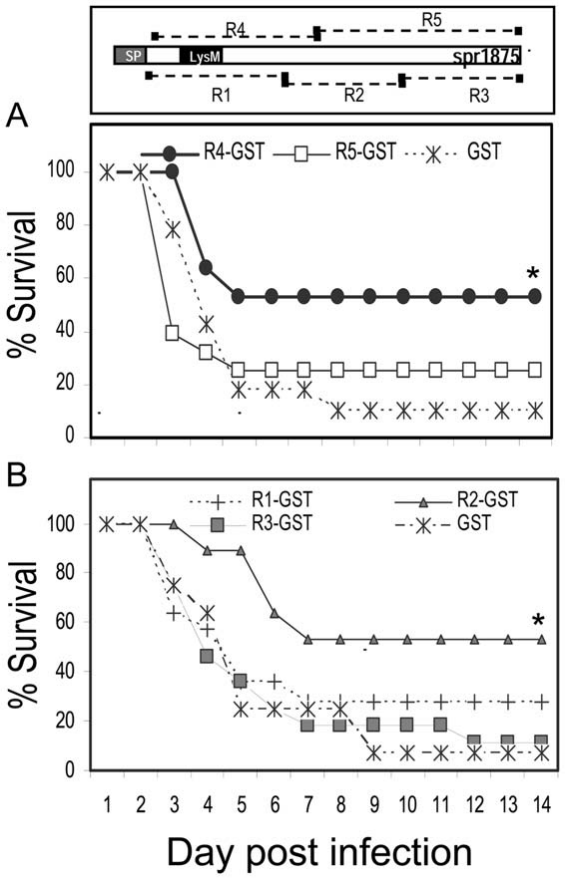
Protection induced by immunization with recombinant fragments of the Spr1875 protein. The diagram illustrates the position of the various fragments relative to the whole protein. (A) Protection induced by R5-GST in comparison with R4-GST. Groups of CD1 mice were immunized with R4-GST, R5-GST or with GST, used as a negative control, and challenged with the D39 strain (1×10^5^ CFU). Results represent cumulative data from 3 experiments. A total number of 28 animals were used for each group. (B) Groups of CD1 mice were immunized with R1-GST, R2-GST, R3-GST or GST used as a negative control, and challenged with the D39 strain (1×10^5^ CFU). Results represent data from one experiment involving 14 animals per group. *, statistically different (p<0.05) from D39 strain, as assessed by Kaplan-Meier estimator of survival.

We next hypothesized that antibodies directed against the R5 portion of the molecule were non-protective. To test this hypothesis, animals were immunized with R5-GST, R4-GST or with GST and challenged with the D39 strain as described above. Indeed, in contrast with the results obtained with R4-GST, R5-GST was totally unable to confer immunoprotection (Fig. 6A). Collectively, these data indicated that immunization with the whole Spr1875 protein induced antibodies predominantly directed against immunodominant epitopes located in R5, a portion of the molecule that does not contain protective epitopes.

Since these data indicated that Spr1875 has immunogenic properties that may radically differ from those observed by using its fragments, we recombinantly expressed three fragments of approximately equal length covering the whole Spr1875 protein. We designated these fragments as R1-GST, R2-GST and R3-GST (Fig. 6, upper panel). The complete sequences of the fragments are provided in Table S3 in the supplemental material. As can be seen in Figure 6B, immunization with R2-GST, but not R1-GST or R3-GST, was protective, and the degree of protection was similar to that of R4-GST immunization. Thus, R4 and R2, but not other portions of the Spr1875 molecule, appeared to contain protective epitopes.

## Discussion

The present study describes the identification and characterization of a novel protein antigen, Spr1875, and of recombinant fragments encompassing the length of this protein. Spr1875 was found to elicit antibodies in the course of human pneumococcal infection, since it was identified by the ability of its R4 fragment to bind antibodies in a convalescent serum. Moreover, further data indicated that the R4 fragment is recognized by a high proportion of sera from patients recovering from pneumococcal disease. Little is presently know on the biological function of Spr1875. The *spr1875* gene was previously found to be strongly upregulated by the VicR component of the VicRK two-component regulatory system together with three other genes encoding one known virulence factors (*pspA*) and putative membrane (*spr0709*) and cell wall (*spr0096*) proteins [9]. Interestingly, Spr0096 and Spr1875 are the only two proteins in *S. pneumoniae* to contain LysM peptidoglycan-binding motifs. Several LysM proteins are known to be virulence factors and/or protective antigens of human bacterial pathogens and some function as adhesins [10]. For example, *Staphylococcus aureus* produces five LysM proteins, which are all involved in virulence [10], [11]. Similar to Spr1875, protein Sip of *Streptococcus agalactiae*, which is considered a promising candidate for an *S. agalactiae* vaccine [12], [13], contains one N-terminal LysM domain.

In the first part of this study, we focused on the expression of the *spr1875* gene and on the impact of Spr1875 upon pneumococcal virulence. Flow cytometry assays demonstrated that Spr1875 is displayed on the surface of pneumococcal strains belonging to different serotypes, although the presence of the polysaccharide capsule largely masked the antigen. This is consistent with other studies reporting that the pneumococcal capsule may mask to varying degrees several surface proteins and adhesins [14], [15]. Moreover, we found that the *spr1875* gene was remarkably conserved amongst serotypes and showed a low degree of polymorphism. Virulence experiments demonstrated that Spr1875 is required for *in vivo* pneumococcal growth, as evidenced by the decreased ability of a Δ*spr1875* deletion mutant to produce lethal infection in mice. The effects of such deletion were marked and equalled or exceeded those observed with a Δ*pspA* deletion mutant. Therefore our data indicate that Spr1875 may behave as an important virulence factor, although further studies are clearly needed to elucidate the function of this protein.

In the second part of the study, we focused on the immunoprotective activity of Spr1875 and on its potential use as a component of a protein-based vaccine. After immunization with the recombinant R4 fragment, we observed significant protection against lethal pneumococcal infection as 50–70% of R4-GST-immunized mice were protected, while only 10–20% of GST-immunized animals survived. In contrast, the whole Spr1875 protein was devoid of immunoprotective activity. This prompted us to analyze in greater detail the nature of the antibody response induced by whole Spr1875. It was found that 80–90% of the reactivity of serum antibodies in Spr1875-immunized animals was directed against the C-terminal portion of the molecule located outside of the R4 fragment. Therefore, only low levels of anti-R4 antibodies were generated by Spr1875 immunization. Moreover, we did not observe protective effects after immunization with a recombinant fragment (R5-GST) encompassing the whole length of the C terminal fragment external to R4. Thus, lack of immunoprotection by the whole Spr1875 protein was linked to the production of antibodies predominantly directed against non-protective, immunodominant epitopes located outside of the R4 fragment. On the contrary, immunoprotection was associated with the induction of anti-R4 antibodies.

Little is known on the molecular factors that determine the immunodominance of some epitopes over others, although this phenomenon is known to occur after immunization with virtually any protein antigen [16]. It is generally thought that antigens bearing immunodominant epitopes are attractive vaccine candidates. However the opposite may be true. As recently noted [16], from the pathogen standpoint it would be useful if the host antibody response was directed against the non-protective portion of a virulence factor containing potentially protective epitopes. In other words, immunodominance of some protein regions may reflect an immune evasion mechanism. In this case, it would be appropriate, in terms of vaccine development, to redirect the response against the non-immunodominant, protective portion of an antigen, by synthesizing protein fragments that don't incorporate the immunodominant epitopes. This seemed to be the case using Rib and a proteins of *S. agalactiae* and the M antigens of *S. pyogens*, in which non immunodominant regions proved to be of particular interest as vaccine components [16]–[18].

Interestingly, in the present study, another Spr1875 fragment (designated as R2) showed, in addition to R4, protective activity against pneumococcal infection after immunization. Since the R2 and R4 fragments overlap in a relatively short (54 aa long) portion of the Spr1875 protein, our data raises the possibility that this overlap region contains the protective epitope(s). Further studies are clearly needed to test this hypothesis. Our data showing that transfer of anti-R4 antibodies protected mice from lethal challenge against the encapsulated D39 strain could, at first glance, appear in contradiction with the inability of such antibodies to bind to the surface of *in vitro* grown D39, due to the masking effect of the capsule. However, it is likely that *in vivo*, at least temporarily or in specific host microenvironments, capsular material is shed from the bacterial surface and/or capsule expression is down-regulated, resulting in exposure of underlying antigens. In support of this notion, several surface pneumococcal antigens whose ability to interact with host components is well documented, are inaccessible to specific antibodies when grown *in vitro* [15].

In conclusion, we have identified a novel immunogenic surface protein with an essential role in virulence. Although complete protection was not observed, these data indicate that selected fragments of the 1875 protein may help, in conjunction with other antigens, in the development of effective vaccines based on pneumococcal proteins.

## Materials and Methods

### Selection of the R4 fragment from the phage display library

The R4 fragment was selected from a genomic pneumococcal phage displayed library using previously described methods [6].

### Pneumococcal strains

The following strains were used: 1) the encapsulated serotype 2 D39 strain [19] and its rough derivative R6 [20]; 2) the unencapsulated mutants Δ-D39, Δ-Tigr4, Δ-23F-Spain-1, and Δ-19F-Taiwan-14, which were kindly provided by Vega Masignani (Novartis Vaccines and Diagnostics s.r.l., Siena, Italy). The D39 deletion mutants Δ*pspA* and Δ*spr1875* (deleted for, respectively, *pspA* (*spr0121*) and *spr1875)* were constructed using a previously described procedure [21]–[23]; see also Text S1 and Tables S2, S3, S4, and S5. Briefly, we first amplified by PCR an antibiotic resistance gene and the 3′ and 5′ genomic regions adjacent to the gene of interest. Next, these fragments were assembled by PCR and the resulting product was used to transform wild type D39 (relevant primers are reported in Table S4 in supplemental materials). The mutants were verified by sequencing (Table S5 in supplemental materials). All bacteria were grown at 37°C in Todd-Hewitt broth (THB, Oxoid). When necessary, chloramphenicol (3 µg/ml) and erythromycin (1 µg/ml) were added. Neither the Δ*pspA*, nor the Δ*spr1875* strain differed from the wild-type D39 strain in its ability to grow in Todd-Hewitt broth.

### Immunization and challenge

To study the protective activity of recombinant proteins, CD1 mice (5 wk old) were immunized intraperitoneally with 50 µg of recombinant proteins fused to glutathione S-transferase (GST) in complete (first injection) or incomplete (second and third injections) Freund's adjuvant emulsions (0.2 ml) on day 0, 14, and 28. Control animals received GST only. The use of complete Freund's adjuvant in the first immunization was justified by our previous observations that high titered sera were more consistently obtained with this adjuvant, as compared to other less “inflammatory” adjuvants such as alum. However, care was taken to minimize discomfort to the animals by injecting a low volume (0.1 ml containing 0.05 mg of mycobacteria) of the oily component of the emulsion and by using sterile solutions and techniques to prepare it. Under these conditions no significant abdominal distension or complications at the injection site were observed throughout the experimental period. Three weeks after the last immunization mice were challenged i.v. with an approximately 90% lethal dose of D39 *S. pneumoniae* strain (1×10^5^ CFU) and monitored for up to14 days. To determine the virulence of deletion mutants pneumococcal strains were grown to mid log phase (OD_600_ = 0.4). Bacteria were washed, resuspended in PBS and plated for colony counts. Eight-week old CD1 mice were inoculated intravenously with 0.1 ml of suspensions containing the indicated bacterial doses. Signs of disease (e.g. rough hair, decreased mobility, lethargy) and lethality were recorded daily for 14 days and animals showing signs of irreversible disease were humanely euthanized. Overwhelming *S. pneumoniae* infection was confirmed as the cause of death by culturing the organs of moribund animals.

For the passive protection experiment, each 8-week-old CD1 mouse received i.p. 50 µl of pooled sera from R4-GST-immunized or GST-immunized mice. Four hours later, mice from both groups were challenged i.v. with the D39 strain and survival was monitored as described above. All *in vivo* experiments were conducted at the animal facilities of the Metchnikoff Department of the University of Messina according to the European Union guidelines for the handling of laboratory animals and were approved by the relevant local (Comitato Etico per la Sperimentazione Animale) and national (Istituto Superiore di Sanità Permit Numbers: 121/2007 - B) authorities.

### Flow cytometry immunofluorescence and western blot analysis

*S. pneumoniae* strains grown to the early-log phase (OD_600_ = 0.2) were harvested by centrifugation, washed three times with PBS and blocked for 20 min at 20°C with PBS containing 2% fetal calf serum (PBS-FCS). Mouse antisera were diluted 1 100 in PBS-FCS and incubated with bacterial cells for 40 min at 4°C. Phycoerythrin-conjugated goat anti-mouse IgG (Jackson Immunoresearch), diluted 1 50 was then added to the cells and incubated at 4°C for additional 30 min. Bacteria were then washed, fixed by paraformaldehyde and analyzed with an LSR Flow Cytometer using the CellQuest software (both from BD Biosciences). Western blots were performed using anti-R4 sera on bacterial cell lysates exactly as described [6].

### Production of recombinant fragments and antisera

The R4 recombinant fragment was amplified from a phage clone and subcloned into the bacterial expression vector pGEX-SN, a previously described expression vector [24] to produce pGEX-SNR4 that allows the expression of recombinant proteins as fusions to GST. Similarly, to produce the GST fusion fragments R1 (117aa; E_24_-S_140_), R2 (124aa; Q_141_-K_264_), R3 (116aa; S_265_-G_380_), and R5 (186aa; T_195_-G_380_) of the Spr1875 protein, or to produce the whole Spr1875 protein fused to GST, the corresponding DNA sequences were amplified from the R6 genome and cloned into pGEX-SN. After induction of the fusion proteins, recombinant fragments were purified from the soluble lysate of bacterial cells by affinity chromatography [6]. Recombinant GST, to be used as a control, was also produced using the same procedures. The whole Spr1875 protein was also produced as a polypeptide fused to a biotinylable peptide tag (BIO) in *Escherichia coli* JM109, using the PinPoint Xa-1 Vector (Promega) according to manufacturer's instructions. After the induction of fusion protein, this was purified from the cytoplasm of bacterial cells by affinity chromatography using the PinPointXa protein purification system (Promega). Recombinant chloramphenicol acetyl transferase fused to biotin (CAT-BIO), to be used as a negative control, was produced and purified using the same methods.

### Determination of R4-specific serum antibody titers

Anti-R4 serum antibody titers in immunized mice were measured by a previously described ELISA method [25]. Briefly, wells of microtiter plates were sensitized with R4-GST fusion protein (5 µg/ml). Mouse sera were serially diluted with antibody buffer containing 25 µg/ml of GST to block non-specific anti-GST antibodies. Serial serum dilutions were reacted for 2 h at 37°C before the addition of a 1 5,000 dilution of goat anti-mouse polyvalent IgG conjugated to alkaline phosphatase (Sigma). Plates were then developed, as described [25]. Antibody titers in human sera were measured by a similar ELISA assay, except that sera were diluted in plain antibody buffer (i.e. without GST) and an anti-human, instead of an anti-mouse, anti-IgG conjugate was used.

### Production of CCR6

To obtain anti-pneumococcal immune sera, to be used as positive controls, a group of mice was immunized with a choline binding proteins-enriched fraction designated as CCR6. CCR6 was obtained from strain R6 cells grown to the early exponential phase (OD_600_ = 0.2), washed with phosphate buffered saline (PBS; pH 7.2), and incubated in the presence of 2% choline chloride (Sigma) at 20°C for 10 minutes. The supernatant was dialyzed, concentrated and used as described above to immunize mice using 50 µg (total protein content) for each immunization.

### Inhibition ELISA

These experiments were performed to assess the ability of recombinant protein fragments to inhibit the reactivity of anti-Spr1875 sera against the homologous antigen. To this end, mouse sera raised against Spr1875-BIO were reacted against Spr1875-GST. An anti-Spr1875-BIO serum pool (diluted 1 200 in PBS-FCS) was added to microtiter wells sensized with Spr1875-GST (1 µg/ml in PBS) in the presence and in the absence of the indicated concentrations of inhibitors (i.e. R4-GST and R5-GST). After washing with PBS-FCS, alkaline phosphatase-conjugated goat anti-mouse IgG (Sigma) was added at a 1 5000 dilution followed by *p*-nitrophenyl phosphate disodium salt (Sigma). Percent of inhibition was calculated by comparing the absorbance value of wells with and without the inhibitors.

## Supporting Information

Figure S1 **Lack of Spr1875 in cell lysates from the Δ** ***spr1875*** **pneumococcal strain.** D39 or Δ*spr1875* cell lysates were loaded on a polyacrylamide gel and developed with anti-R4 mouse serum (1 1,000). The arrow indicates the 40 kDa Spr1875 protein in the D39, but not in the *Δspr1875*,cell lysate.
(TIF)

Figure S2 **Protection induced by R4 immunization is mediated by serum antibodies.** Two groups of 16 mice each were given i.p. 50 µl of serum pools from animals immunized with, respectively, GST (anti-GST) or R4-GST (anti-R4-GST). After 4 h both groups of animals were challenged i.v., with D39 (1×10^5^ CFU). *, statistically different (p<0.05) from animals given anti-GST serum as assessed by Kaplan-Meier estimator of survival.
(TIF)

Figure S3 **Lack of protection induced by immunization with recombinant Spr1875 protein fused to a GST tag (Spr1875-GST).** (A) Groups of CD1 mice were immunized with Spr1875-GST or with GST, used as a negative control, and challenged with the D39 strain (1×10^5^ CFU). Results represent data from one experiment involving 8 animals per group.
(TIF)

Table S1 **Anti-R4 Elisa titers of sera from patients convalescing from pneumococcal infection.**
(DOC)

Table S2 **Alignment of 27** ***S. pneumoniae*** **strain sequences available in DNA database using the ClustalW software (** http://www.ebi.ac.uk/Tools/clustalw2/index.html **).**
(DOC)

Table S3 **Amino acid sequences of Spr1875 fragments.**
(DOC)

Table S4 **Primers used in the construction of Δ** ***pspA*** **and Δ** ***spr1875*** **deletion mutants.**
(DOC)

Table S5 **Genomic replacement sequences for Δ** ***pspA*** **and Δ** ***spr1875*** **deletion mutants.**
(DOC)

Text S1 **Supplemental Materials and Methods.**
(DOC)
