# Supplementary figures and images for: Protective Activity of Streptococcus pneumoniae Spr1875 Protein Fragments Identified Using a Phage Displayed Genomic Library
Source: PLoS One. 2012 May 3;7(5):e36588. doi: 10.1371/journal.pone.0036588 (PMC3343019; doi:10.1371/journal.pone.0036588)

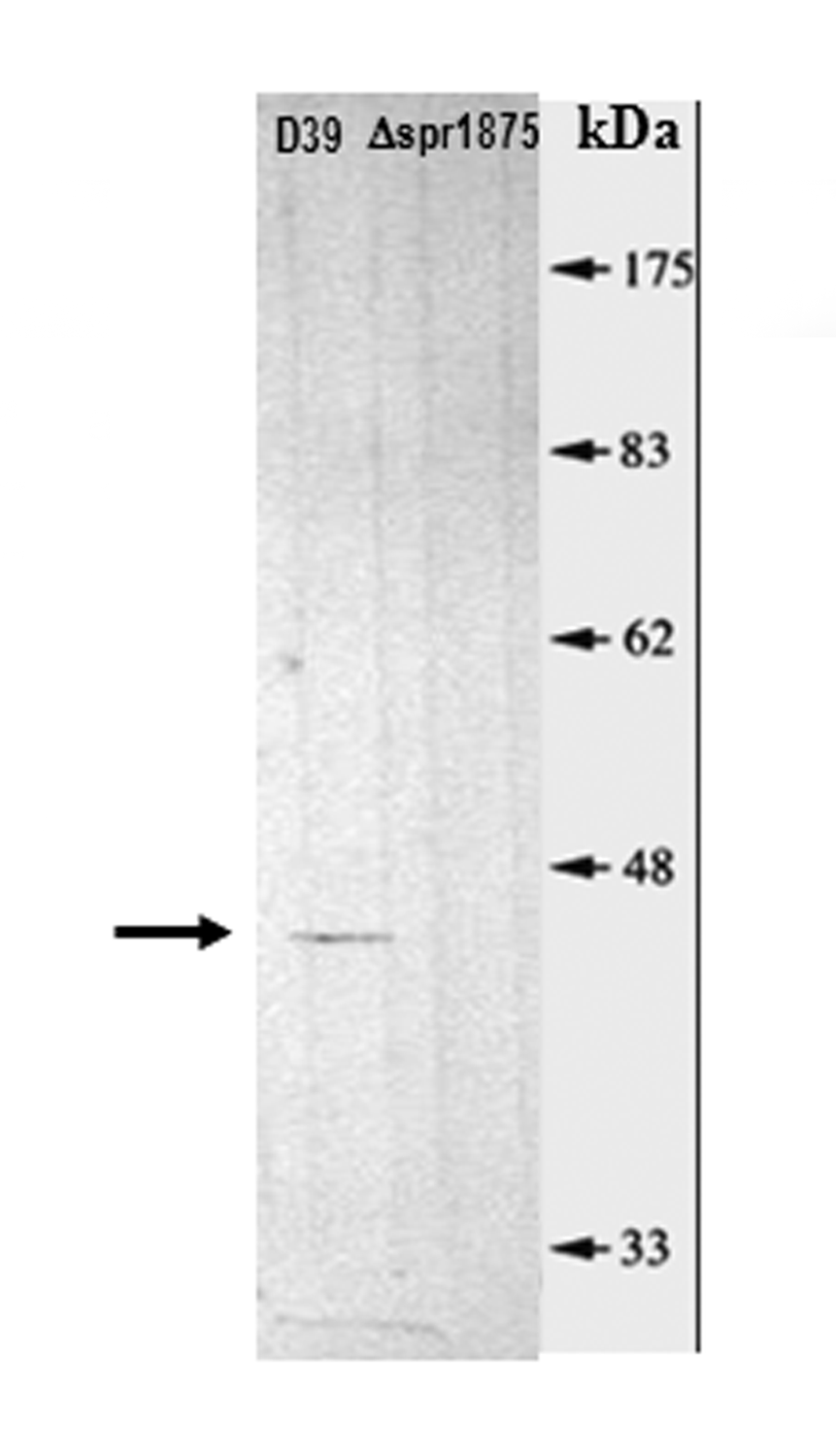

Supplement: Figure S1 — Lack of Spr1875 in cell lysates from the Δ spr1875 pneumococcal strain. D39 or Δspr1875 cell lysates were loaded on a polyacrylamide gel and developed with anti-R4 mouse serum (1 1,000). The arrow indicates the 40 kDa Spr1875 protein in the D39, but not in the Δspr1875,cell lysate. (TIF) [file pone.0036588.s001.tif]

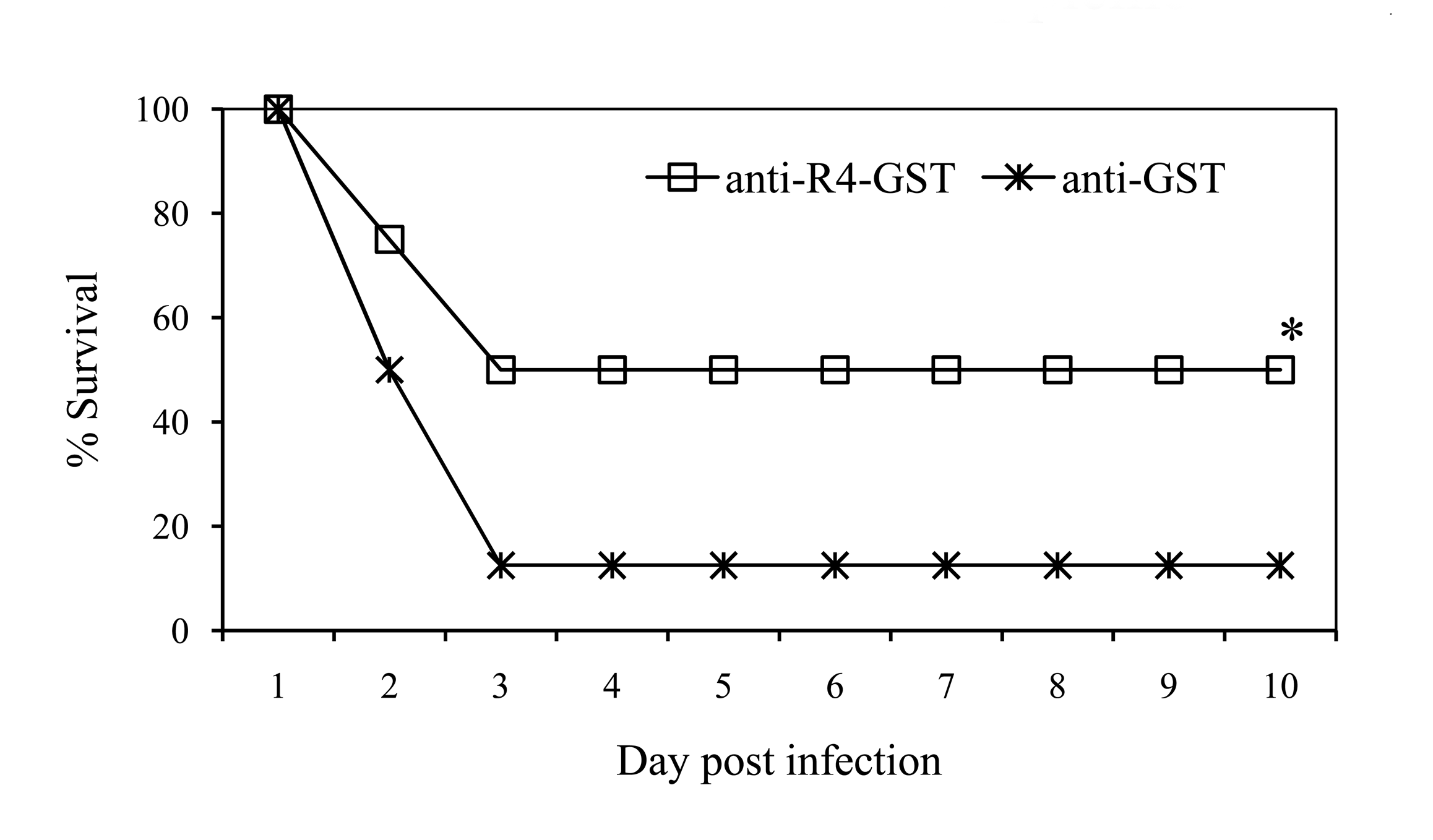

Supplement: Figure S2 — Protection induced by R4 immunization is mediated by serum antibodies. Two groups of 16 mice each were given i.p. 50 µl of serum pools from animals immunized with, respectively, GST (anti-GST) or R4-GST (anti-R4-GST). After 4 h both groups of animals were challenged i.v., with D39 (1×10^5^ CFU). *, statistically different (p<0.05) from animals given anti-GST serum as assessed by Kaplan-Meier estimator of survival. (TIF) [file pone.0036588.s002.tif]

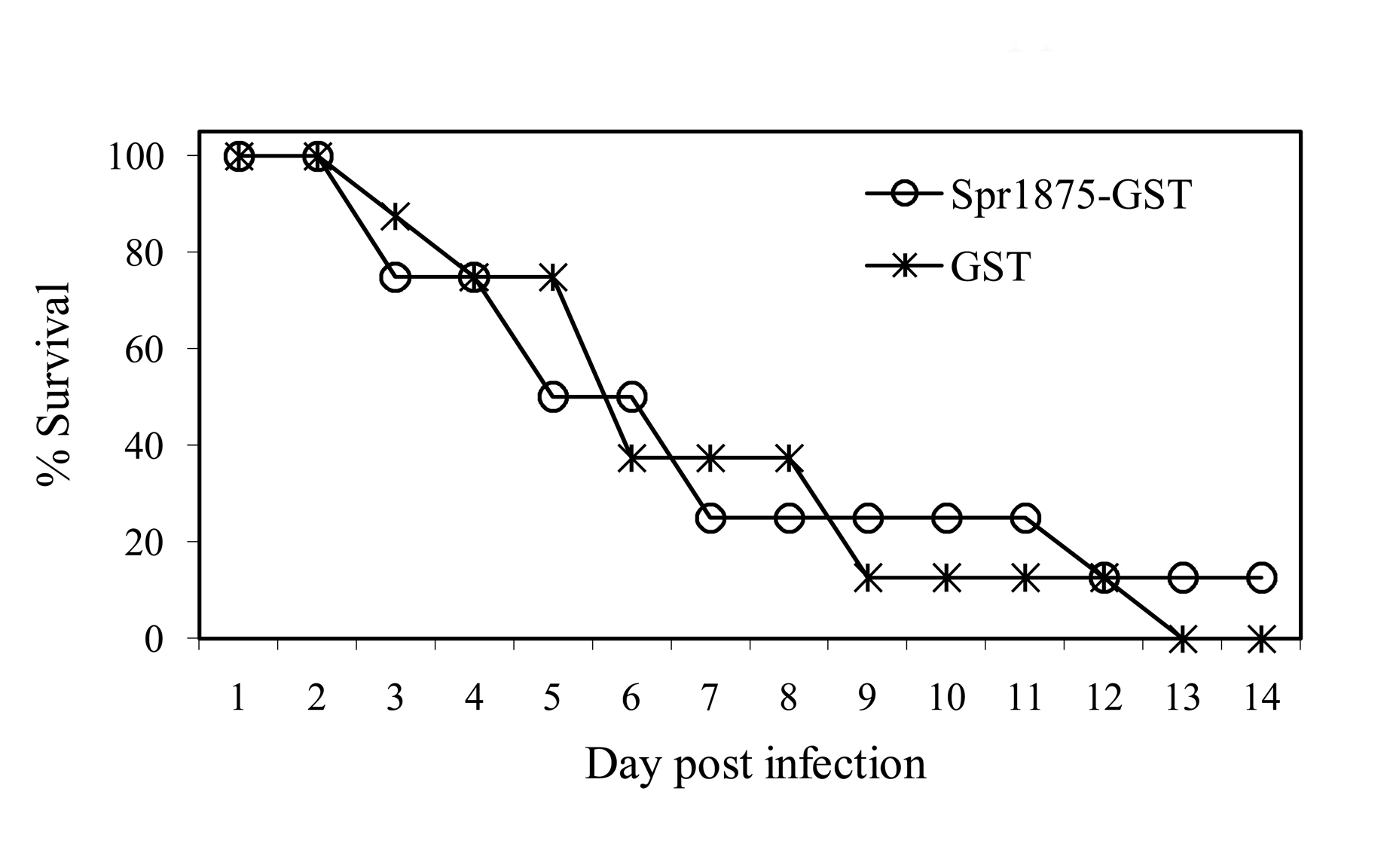

Supplement: Figure S3 — Lack of protection induced by immunization with recombinant Spr1875 protein fused to a GST tag (Spr1875-GST). (A) Groups of CD1 mice were immunized with Spr1875-GST or with GST, used as a negative control, and challenged with the D39 strain (1×10^5^ CFU). Results represent data from one experiment involving 8 animals per group. (TIF) [file pone.0036588.s003.tif]
