# Supplementary material for: Protective Activity of Streptococcus pneumoniae Spr1875 Protein Fragments Identified Using a Phage Displayed Genomic Library
Source: PLoS One. 2012 May 3;7(5):e36588. doi: 10.1371/journal.pone.0036588 (PMC3343019; doi:10.1371/journal.pone.0036588)
Supplement: Table S1 — Anti-R4 Elisa titers of sera from patients convalescing from pneumococcal infection. (DOC) [file pone.0036588.s004.doc]

Anti-R4 Elisa titers of sera from patients convalescing from pneumococcal infection

| **Serum source**  Control serum  Control serum  Control serum  Control serum  Control serum  Control serum  Patient’s serum  Patient’s serum  Patient’s serum  Patient’s serum  Patient’s serum  Patient’s serum  Patient’s serum  Patient’s serum  Patient’s serum  Patient’s serum | **Id**  189F5  192Q7  191C2  189A1  189F5  189B3  79I10  105E3  132R7  144R6  155H3  156O9  165L1  168S10  188S7  168N7 | **Diagnosis**  adenovirus infection  enteritis  skin exanthema  stomatitis  respiratory infection  infectious mononucleosis  pleuritis  pleuritis  bronchopneumonia  septic arthritis  pneumonia  pneumonia  pneumonia  pleuritis  pneumonia  pneumonia | **α-GST titer**  <200^a^  <200  <200  <200  <200  <200  <200  <200  <200  <200  <200  <200  <200  <200  <200  <200 | **α-R4 GST titer**  <200  <200  <200  <200  <200  <200  1:800  <200  1:400  1:1600  1:800  1:6400  1:400  1:400  1:400  <200 |
| --- | --- | --- | --- | --- |

**^a^**)Wells of microtiter plates were sensitized with recombinant GST or R4-GST (5μg/ml). Serial dilutions of human sera (from 1:200 to 12,800) were reacted for 2h at 37°C before the addition of anti-human polyvalent IgG conjugated to alkaline phosphatase. A_405_ was measured after the addition of p-nitrophenyl phosphate. The titer was determined as the highest serum dilution producing an A_405_ >0.2. Control sera were from age-matched patients suffering from infections caused by various non-pneumococcal agents. Patients’ sera were from patients with culture-proven pneumococcal disease. Serum samples were obtained at 15-44 days from the onset of disease.
