# Supplementary material for: Protective Activity of Streptococcus pneumoniae Spr1875 Protein Fragments Identified Using a Phage Displayed Genomic Library
Source: PLoS One. 2012 May 3;7(5):e36588. doi: 10.1371/journal.pone.0036588 (PMC3343019; doi:10.1371/journal.pone.0036588)
Supplement: Table S2 — Alignment of 27 S. pneumoniae strain sequences available in DNA database using the ClustalW software ( http://www.ebi.ac.uk/Tools/clustalw2/index.html ). (DOC) [file pone.0036588.s005.doc]

**Alignment of 27 *S. pneumoniae* strain sequences available in DNA database using the ClustalW software** (<http://www.ebi.ac.uk/Tools/clustalw2/index.html>).

All strains belonged to one of 14 sequence groups (I-XIV)

Strains grouped as I to XIV are listed below:

**Group I**: D39, R6; **Group II**: Canada MDR_19F, Taiwan 19F-14, Canada MDR_19A, TCH8431/19A; **Group III**: SP23-BS72; **Group IV**: CDC1087-00; **Group V**: CDC0288-04; **Group VI**; Hungary19A-6; **Group VII**: SP14-BS69, CCRI 1974, CCRI 1974M2; **Group VIII**: SP19-BS75; **Group IX**; SP3-BS71; **Group X**: TIGR4, SP18-BS74, CDC3059-06; **Group XI**: SP11-BS70, MLV-016, G54, AP200; **Group XII**: SP9-BS68, SP195, GA17570; **Group XIII**: P1031; **Group XIV**:SP6-BS73.

I_ MKKRMLLASTVALSFAPVLATQAEEVLWTARSVEQIQNDLTKTDNKTSYTVQYGDTLSTI 60

II_ MKKRMLLASTVALSFAPVLATQAEEVLWTARSVEQIQNDLTKTDNKTSYTVQYGDTLSTI 60

III_ MKKRMLLASTVALSFAPVLATQAEEVLWTARSVEQIQNDLTKTDNKTSYTVQYGDTLSTI 60

VI_ MKKRMLLASTVALSFAPVLATQAEEVLWTARSVEQIQNDLTKTDNKTSYTVQYGDTLSTI 60

XII_ MKKRMLLASTVALSFAPVLATQAEEVLWTARSVEQIQNDLTKTDNKTSYTVQYGDTLSTI 60

V_ MKKRMLLASTVALSFAPVLATQAEEVLWTARSVEQIQNDLTKTDNKTSYTVQYGDTLSTI 60

IX_ ----MLLASTVALSFAPVLATQAEEVLWTARSVEQIQNDLTKTDNKTSYTVQYGDTLSTI 56

IV_ MKKRMLLASTVALSFAPVLATQAEEVLWTARSVEQIQNDLTKTDNKTSYTVQYGDTLSTI 60

X_ MKKRMLLASTVALSFAPVLATQAEEVLWTARSVEQIQNDLTKTDNKTSYTVQYGDTLSTI 60

VII_ MKKRMLLASTVALSFAPVLATQAEEVLWTARSVEQIQNDLTKTDNKTSYTVQYGDTLSTI 60

XI_ MKKRMLLASTVALSFAPVLATQAEEVLWTARSVEQIQNDLTKTDNKTSYTVQYGDTLSTI 60

VIII_ MKKRMLLASTVALSFAPVLATQAEEVLWTARSVEQIQNDLTKTDNKTSYTVQYGDTLSTI 60

XIII_ MKKRMLLASTVALSFAPVLATQAEEVLWTARSVEQIQNDLTKTDNKTSYTVQYGDTLSTI 60

XIV_ ----MLLASTVALSFAPVLATQAEEVLWTARSVEQIQNDLTKTDNKTSYTVQYGDTLSTI 56

************************************************************

I_ AEALGVDVTVLANLNKITNMDLIFPETVLTTTVNEAEEVTEVEIQTPQADSSEEVTTATA 120

II_ AEALGVDVTVLANLNKITNMDLIFPETVLTTTVNEAEEVTEVEIQTPQADSSEEVTTATA 120

III_ AEALGVDVTVLANLNKITNMDLIFPETVLTTTVNEAEEVTEVEIQTPQADSSEEVTTATA 120

VI_ AEALGVDVTVLANLNKIINMDLIFPETVLTTTVNEAEEVTEVEIQTPQADSSEEVTTATA 120

XII_ AEALGVDVTVLANLNKITNMDLIFPETVLTTTVNEAEEVTEVEIQTPQADSSEEVTTATA 120

V_ AEALGVDVTVLANLNKITNMDLIFPETVLTTTVNEAEEVTEVEIQTPQADSSEEVTTATA 120

IX_ AEALGVDVTVLANLNKITNMDLIFPETVLTTTVNEAEEVTEVEIQTPQADSSEEVTTATA 116

IV_ AEALGVDVTVLANLNKITNMDLIFPETVLTTTVNEAEEVTEVEIQTPQADSSEEVTTATA 120

X_ AEALGVDVTVLANLNKITNMDLIFPETVLTTTVNEAEEVTEVEIQTPQADSSEEVTTATA 120

VII_ AEALGVDVTVLANLNKITNMDLIFPETVLTTTVNEAEEVTEVEIQTPQADSSEEVTTATA 120

XI_ AEALGVDVTVLANLNKITNMDLIFPETVLTTTVNEAEEVTEVEIQTPQADSSEEVTTATA 120

VIII_ AEALGVDVTVLANLNKITNMDLIFPETVLTTTVNEAEEVTEVEIQTPQADSSEEVTTATA 120

XIII_ AEALGVDVTVLANLNKITNMDLIFPETVLTTTVNEAEEVTEVEIQTPQADSSEEVTTATA 120

XIV_ AEALDVDVTVLANLNKITNMDLIFPETVLTTTVNEAEEVTEVEIQTPQADSSEEVTTATA 116

******.************ ********************************************

I_ DLTTNQVTVDDQTVQVADLSQPIAEAPKEVASSSEVTKTVIASEEVAPSTGTSVPEEQTA 180

II_ DLTTNQVIVDDQTVQVADLSQPIAEAPKEVASSSEVTKTVIASEEVAPSTGTSVPEEQTA 180

III_ DLTTNQVTVDDQTVQVADLSQPIAEAPKEVASSSEVTKTVIASEEVAPSTGTSVPEEQTA 180

VI_ DLTTNQVTVDDQTVQVADLSQPIAEAPKEVASSSEVTKTVIASEEVAPSTGTSVPEEQTA 180

XII_ DLTTNQVTVDVQTVQVADLSQPIAEAPKEVASSSEVTKTVIASEEVAPSTGTSVPEEQTA 180

V_ DLTTNQVTVDDQTVQVADLSQPIAEAPKEVASSSEVTKTVIASEEVAPSTGTSVPEEQTA 180

IX_ DLTTNQVTVDDQTVQVADLSQPIAEAPKEVASSSEVTKTVIASEEVAPSTGTSVPEEQTA 176

IV_ DLTTNQVTVDDQTVQVADLSQPIAEAPKEVASSSEVTKTVIASEEVAPSTGTSVPEEQTT 180

X_ DLTTNQVTVDDQTVQVADLSQPIA----------EVTKTVIASEEVAPSTGTSVPEEQTT 170

VII_ DLTTNQVTVDDQTVQVADLSQPIAEAPKEVASSSEVTKTVIASEEVAPSTGTSVPEEQTT 180

XI_ DLTTNQVTVDDQTVQVADLSQPIA----------EVTKTVIASEEVAPSTGTSVPEEQTT 170

VIII_ DLTTNQVTVDDQTVQVADLSQPIAEAPKEVASSSEVTKTVIASEEVAPSTGTSVPEEQTA 180

XIII_ DLTTNQVTVDDQTVQVADLSQPIAEAPKEVASNSEVAETVTAAEEVALSTDSTTPEGQPA 180

XIV_ DLTTNQVTVDDQTVQVADLSQPIAEAPKEVASNSEVAETVTAAEEVALSTDSTTPEGQPA 176

********* ** ************* **::** *:**** **.::.** *.:**

I_ ETSSAVAEEAPQETTPAEKQETQTSPQAASAVEATT------------TSSEAKEVASSN 228

II_ ETSSAVAEEAPQETTPAEKQETQASPQAASAVEVTT------------TSSEAKEVASSN 228

III_ ETSSAVAEEAPQETTPGEKQETQASPQAASAVEATT------------TSSEAKEVASSN 228

VI_ ETTRPVEEATPQETTPAEKQETQASPQAASAVEATT------------TSSEAKEVASSN 228

XII_ ETTRPVEEATPQETTPAKKQETQASPQAASAVEATT------------TSSEAKEVASSN 228

V_ ETTRPVEEATPQETTPAEKQETQASPQAASVVEATT------------TSSEAKEVASSN 228

IX_ ETTRPVEEATPQETTPAEKQETQASPQAALAVEATT------------TSSEAKEVASSN 224

IV_ ETTRPVEEATPQETTPAEKQETQASPQAASAVEVTT------------TSSEAKEVASSN 228

X_ ETTRPVEEATPQETTPAEKQETQASPQAASAVEVTT------------TSSEAKEVASSN 218

VII_ ETTRPVEEATPQETTPAEKQETQASPQAASAVEVTT------------TSSEAKEVASSN 228

XI_ ETTRPVEEATPQETTPAEKQETQASPQAASAVEVTT------------TSSEAKEVASSN 218

VIII_ ETSSAVAEEAPQETTPAEKQETQVSSQTESAVEATTMPVEEKATETTATSSEAKEVASSN 240

XIII_ ETTSPVEEVAPQATTLAEKQETQVSSQTESAVEATTMPVEEKATEATTTSSEAKEVASSN 240

XIV_ ETTSPVEEVAPQATTLAEKQETQVSSQTESAVEATTMPVEEKATETTATSSEAKEVASSN 236

****: .* * :** ** .:*****.*.*: .**.** **************

I_ GATAAVSTYQPEETKIISTTYEAPAAPDYAGLAVAKSENAGLQPQTAAFKEEIANLFGIT 288

II_ GATAAVSTYQPEETKIISTTYEAPAAPDYAGLAVAKSENAGLQPQTAAFKEEIANLFGIT 288

III_ GATAAVSTYQPEETKIISTTYEAPAAPDYAGLAVAKSENAGLQPQTAAFKEEIANLFGIT 288

VI_ GATAAVSTYQPEETKVISTTYEAPAAPDYAGLAVAKSENAGLQPQTAAFKEEIANLFGIT 288

XII_ GATAAVSTYQPEETKVISTTYEAPAAPDYAGLAVAKSENAGLQPQTAAFKEEIANLFGIT 288

V_ GATAAVSTYQPEETKVISTTYEAPAAPDYAGLAVAKSENAGLQPQTAAFKEEIANLFGIT 288

IX_ GATAAVSTYQSEETKVISTTYEAPAAPDYAGLAVAKSENAGLQPQTAAFKEEIANLFGIT 284

IV_ GATAAVSTYQPEETKIISTTYEAPAAPDYAGLAVAKSENAGLQPQTAAFKEEIANLFGIT 288

X_ GATAAVSTYQPEETKIISTTYEAPAAPDYAGLAVAKSENAGLQPQTAAFKEEIANLFGIT 278

VII_ GATAAVSTYQPEETKIISTTYEAPAAPDYAGLAVAKSENAGLQPQTAAFKEEIANLFGIT 288

XI_ GATAAVSTYQPEETKVISTTYEAPAAPDYAGLAVAKSENAGLQPQTAAFKEEIANLFGIT 278

VIII_ GATAAVSTYQPEETKTISTTYEAPAAPDYAGLAVAKSENAGLQPQTAAFKEEIANLFGIT 300

XIII_ GATAAVSTYQPEETKIISTTYEAPAAPDYAGLAVTKSENAGLQPQTAAFKEEIANLFGIT 300

XIV_ GATAAVSTYQPEETKTISTTYEAPAAPDYAGLAVAKSENAGLQPQTAAFKEEIANLFGIT 296

************.**** ******************:***************************

I_ SFSGYRPGDSGDHGKGLAIDFMVPERSELGDKIAEYAIQNMASRGISYIIWKQRFYAPFD 348

II_ SFSGYRPGDSGDHGKGLAIDFMVPERSELGDKIAEYAIQNMASRGISYIIWKQRFYAPFD 348

III_ SFSGYRPGDSGDHGKGLAIDFMVPESSELGDKIAEYAIQNMASRGISYIIWKQRFYAPFD 348

VI_ SFSGYRPGDSGDHGKGLAIDFMVAERSELGDKIAEYAIQNMASRGISYIIWKQRFYAPFD 348

XII_ SFSGYRPGDSGDHGKGLAIDFMVAERSELGDKIAEYAIQNMASRGISYIIWKQRFYAPFD 348

V_ SFSGYRPGDSGDHGKGLAIDFMVAERSELGDKIAEYAIQNMASRGISYIIWKQRFYAPFD 348

IX_ SFSGYRPGDSGDHGKGLAIDFMVPERSELGDKIAEYAIQNMASRGISYIIWKQRFYAPFD 344

IV_ SFSGYRPGDSGDHGKGLAIDFMVPERSELGDKIAEYAIQNMASRGISYIIWKQRFYAPFD 348

X_ SFSGYRPGDSGDHGKGLAIDFMVPERSELGDKIAEYAIQNMASRGISYIIWKQRFYAPFD 338

VII_ SFSGYRPGDSGDHGKGLAIDFMVPEHSELGDKIAEYAIQNMASRGISYIIWKQRFYAPFD 348

XI_ SFSGYRPGDSGDHGKGLAIDFMVPERSELGDKIAEYAIQNMASRGISYIIWKQRFYAPFD 338

VIII_ SFSGYRPGDSGDHGKGLAIDFMVPESSELGDKIAEYAIQNMASRGISYIIWKQRFYAPFD 360

XIII_ SFSGYRPGDSGDHGKGLAIDFMVPERSELGDKIAEYAIQNMASRGISYIIWKQRFYAPFD 360

XIV_ SFSGYRPGDSGDHGKGLAIDFMVPESSELGDKIAEYAIQNMASRGISYIIWKQRFYAPFD 356

*************************.* ************************************

I_ SKYGPANTWNPMPDRGSVTENHYDHVHVSMNG 380

II_ SKYGPANTWNPM-------------------- 360

III_ SKYGPANTWNPMPDRGSVTENHYDHVHVSMNG 380

VI_ SKYGPANTWNPMPDRGSVTENHYDHVHVSMNG 380

XII_ SKYGPANTWNPMPDRGSVTENHYDHVHVSMNG 380

V_ SKYGPANTWNPMPDRGSVTENHYDHVHVSMNG 380

IX_ SKYGPANTWNPMPDRGSVTENHYDHVHVSMNG 376

IV_ SKYGPANTWNPMPDRGSVTENHYDHVHVSMNG 380

X_ SKYGPANTWNPMPDRGSVTENHYDHVHVSMNG 370

VII_ SKYGPANTWNPMPDRGSVTENHYDHVHVSMNG 380

XI_ SKYGPANTWNPMPDRGSVTENHYDHVHVSMNG 370

VIII_ SKYGPANTWNPMPDRGSVTENHYDHVHVSMNG 392

XIII_ SKYGPANTWNPMPDRGSVTENHYDHVHVSMNG 392

XIV_ SKYGPANTWNPMPDRGSVTENHYDHVHVSMNG 388

****************
