# Supplementary material for: Protective Activity of Streptococcus pneumoniae Spr1875 Protein Fragments Identified Using a Phage Displayed Genomic Library
Source: PLoS One. 2012 May 3;7(5):e36588. doi: 10.1371/journal.pone.0036588 (PMC3343019; doi:10.1371/journal.pone.0036588)
Supplement: Table S3 — Amino acid sequences of Spr1875 fragments. (DOC) [file pone.0036588.s006.doc]

**Amino acid sequences of Spr1875 fragments**

**R1** (117aa; E24-S140)

EEVLWTARSVEQIQNDLTKTDNKTSYTVQYGDTLSTIAEALGVDVTVLANLNKITNMDLIFPETVLTTTVNEAEEVTEVEIQTPQADSSEEVTTATADLTTNQVTVDDQTVQVADLS

**R2** (124aa; Q 141-K264)

QPIAEAPKEVASSSEVTKTVIASEEVAPSTGTSVPEEQTAETSSAVAEEAPQETTPAEKQETQTSPQAASAVEATTTSSEAKEVASSNGATAAVSTYQPEETKIISTTYEAPAAPDYAGLAVAK

**R3** (116aa; S265- G380)

SENAGLQPQTAAFKEEIANLFGITSFSGYRPGDSGDHGKGLAIDFMVPERSELGDKIAEYAIQNMASRGISYIIWKQRFYAPFDSKYGPANTWNPMPDRGSVTENHYDHVHVSMNG

**R5** (189aa; Q192- G380)

QETTPAEKQETQTSPQAASAVEATTTSSEAKEVASSNGATAAVSTYQPEETKIISTTYEAPAAPDYAGLAVAKSENAGLQPQTAAFKEEIANLFGITSFSGYRPGDSGDHGKGLAIDFMVPERSELGDKIAEYAIQNMASRGISYIIWKQRFYAPFDSKYGPANTWNPMPDRGSVTENHYDHVHVSMNG
