# Supplementary material for: Protective Activity of Streptococcus pneumoniae Spr1875 Protein Fragments Identified Using a Phage Displayed Genomic Library
Source: PLoS One. 2012 May 3;7(5):e36588. doi: 10.1371/journal.pone.0036588 (PMC3343019; doi:10.1371/journal.pone.0036588)
Supplement: Table S4 — Primers used in the construction of Δ pspA and Δ spr1875 deletion mutants. (DOC) [file pone.0036588.s007.doc]

**Primers used in the construction of Δ*pspA* and Δ*spr1875* deletion mutants**

| **PRODUCT** | **PRIMER** |
| --- | --- |
| ***pspA*-**upstream region | **1**: 5’- TTGGGCAGTAGTGAGAACTG-3’  **2**: 5’-CATCAACAATCACAAATCACTTCAGACTATACTTATATTAAG-3’ |
| ***pspA***-downstream-region | **3**: 5’-CTTCCAAGGAGCTAAAGAGGTGCCGATTAAATTAAAGCATG-3’  **4** : 5’-ATCTTCGGTCGCCGTACAGA-3’ |
| Erythromycin-resistance | **IF188**: 5’-AAGTGATTTGTGATTGTTGATG-3’  **IF189** : 5’ACCTCTTTAGCTCCTTGGAAG-3’ |
| ***spr1875-***upstream region | **1**: 5’-TGAGAGAGTTTATCTTCAAG-3’  **2**: 5’-CCATTAAAAATCAAACAAATTTTCATTCTTTCAAATTCCTTTCA-3’ |
| ***spr1875-***downstream region | **3** **3**:5’-AGATAGGCCTAATGACTGGCTTTTATAACAATGAATGGATAAACC-3’  **4**: 5’:AACGCTCCTGAACCGCCATT-3’ |
| Chloramphenicol-resistance | **IF38** : 5’-ATGAAAATTTGTTTGATTTTTAATGG-3’  **IF39**: 5’- TTATAAAAGCCAGTCATTAGGCCTATCT-3’ |
