# Supplementary material for: Protective Activity of Streptococcus pneumoniae Spr1875 Protein Fragments Identified Using a Phage Displayed Genomic Library
Source: PLoS One. 2012 May 3;7(5):e36588. doi: 10.1371/journal.pone.0036588 (PMC3343019; doi:10.1371/journal.pone.0036588)
Supplement: Table S5 — Genomic replacement sequences for Δ pspA and Δ spr1875 deletion mutants. (DOC) [file pone.0036588.s008.doc]

**Genomic replacement sequences for Δ*pspA* and Δ*spr1875* deletion mutants.**

The primers are highlighted in grey and the resistance cassette is underlined.

**Δ*pspA***

TTGGGCAGTAGTGAGAACTGCCCCGTGCTTTTTTATTTTGAGAAAATATGGAGTTTGTCGTTGAAATTACTTGATTGTATTTTGGACTATCAAGAAAGATTCAATGGAAAAACATGTCAAGTATCAACGAATTATAAGTATTTAGAGACTTTCAAAGTGAATTTTTGCTTGACTGATTTACATCATTTATTTGACTTATACAAAATCACACGAGATTATGCTAGTCAAACAAAACCTGCTATTCAAGCTGGTGTTTTTATTTTAGAAGATTTTAGAAATATCCTATGTACAATGATGTAATCGAAAGGATATCTTTATGTGAGTTTATCGGCGATATTTTCTATTCTAAAATAACAAGTTGTTGCATCGTAGCTAAGGATTTATCTAAAAATACTATGAAATTGGACGTCATATTTTTTGAGGATAGAAATAAAAGATCCGCAGTTTTAGGTTTACGAAGAGACAAAAGCGGAGTATTTAAACCAGTTCCCCTACATTTTACAAGCGCTAAGAAATATGCTAAAGTTCGTAAAACAGATGTGAAAGAAATGAAATGGCTATAAAGATTGTCCGCAGGCTTAAGCTTGCGTTTTTTGTTGTTTAACGAAATTAAAAAACAAGCTCTCTCATCGGAAATGTTTTTATAGGAATGAAGGAAGATGATGCTATGTTTAAAAAGAGAATTGAAAGGGGCAAAAGTAGTATCTTTTATCTATTTTTGGGAGTAGAAGCTTATGATATAGAAATTTGTAACAAAAATGTAATATAAAACACTTGACAAATATTTACGGAGGAGGCTATACTTAATATAAGTATAGTCTGAAGTGATTTGTGATTGTTGATGATAAAATAAGAATAAGAAGAAATAGAAAGAAGTGAGTGATTGTGGGAAATTTAGGCGCACAAAAGAAAAACGAAATGATACACCAATCAGTGCAAAAAAAGATATAATGGGAGATAAGACGGTTCGTGTTCGTGCTGACTTGCACCATATCATAAAAATCGAAACAGCAAAGAATGGCGGAAACGTAAAAGAAGTTATGGAAATAAGACTTAGAAGCAAACTTAAGAGTGTGTTGATAGTGCAGTATCTTAAAATTTTGTATAATAGGAATTGAAGTTAAATTAGATGCTAAAAATTTGTAATTAAGAAGGAGTGATTACATGAACAAAAATATAAAATATTCTCAAAACTTTTTAACGAGTGAAAAAGTACTCAACCAAATAATAAAACAATTGAATTTAAAAGAAACCGATACCGTTTACGAAATTGGAACAGGTAAAGGGCATTTAACGACGAAACTGGCTAAAATAAGTAAACAGGTAACGTCTATTGAATTAGACAGTCATCTATTCAACTTATCGTCAGAAAAATTAAAACTGAATACTCGTGTCACTTTAATTCACCAAGATATTCTACAGTTTCAATTCCCTAACAAACAGAGGTATAAAATTGTTGGGAGTATTCCTTACCATTTAAGCACACAAATTATTAAAAAAGTGGTTTTTGAAAGCCATGCGTCTGACATCTATCTGATTGTTGAAGAAGGATTCTACAAGCGTACCTTGGATATTCACCGAACACTAGGGTTGCTCTTGCACACTCAAGTCTCGATTCAGCAATTGCTTAAGCTGCCAGCGGAATGCTTTCATCCTAAACCAAAAGTAAACAGTGTCTTAATAAAACTTACCCGCCATACCACAGATGTTCCAGATAAATATTGGAAGCTATATACGTACTTTGTTTCAAAATGGGTCAATCGAGAATATCGTCAACTGTTTACTAAAAATCAGTTTCATCAAGCAATGAAACACGCCAAAGTAAACAATTTAAGTACCGTTACTTATGAGCAAGTATTGTCTATTTTTAATAGTTATCTATTATTTAACGGGAGGAAATAATTCTATGAGTCGCTTTTGTAAATTTGGAAAGTTACACGTTACTAAAGGGAATGTAGATAAATTATTAGGTATACTACTGACAGCTTCCAAGGAGCTAAAGAGGTGCCGATTAAATTAAAGCATGTTAAGAACATTTGACATTTTAATTTTGAAACAAAGATAAGGTTCGATTGAATAGATTTATGTTCGTATTCTTTAGGTACCTATCTTATGATTTCAGGAAATGTCATTAAAAAAACGACTCCTTTTCTCTAACTTGAAAAATAGATTAGAGAAAATGGGTTGTTTTATTTATTATAGTTATTTGAATGAAGATAAGAAGAAGGTATACTCACATCATTCACATAATCTGTATATTGACTATAAGTTTTAAAAAACAATTTTTAAGCTCTTCCTTGTCTTCTCTAACCAAGCGTGTTATAATGAATACTGCTCAAGCGACCTTCAATCGTGAAGCACACACGACCTTCAATCGTGAATAAACGAATAGATGGGAGACTTACCATGAGTGATAACTCTAAAACACGTGTTGTCGTGGGGATGAGTGGTGGTGTTGATTCGTCGGTGACGGCTCTTTTGCTCAAGGAGCAGGGCTACGATGTGATCGGTATCTTCATGAAGAACTGGGATGACACAGATGAAAACGGCGTCTGTACGGCGACCGAAGAT

**Δ*spr1875***

TGAGAGAGTTTATCTTCAAGTATTCGGTGCAAGATTTGCTTGTGCGGGTGGCAGAAGATAGAAATCTGGATGTTGAGGTGCTAAATCAGGTGCGTGCCCAGAGTCTGGCTGAGAAGAATGCTCAGGTAGTTTTGATGCCAGGTGCGCGTGAGGTGCTAGCTTGGGCAGACGAATCAGGAATTCAGCAGTTTATATATACTCATAAGGGGAACAACGCTTTTACCATTCTCAAGGACTTGGGGGTGGAATCCTATTTTACAGAGATTTTAACCAGTCAGAGTGGCTTTGTGCGGAAGCCAAGTCCAGAAGCGGCTACCTATCTGCTAGATAAGTATCAGTTGAATTCTGATAATACTTATTATATAGGGGATCGGACTCTGGATGTGGAATTTGCCCAGAATAGTGGGATTCAAAGTATCAACTTTTTAGAGTCTACTTATGAAGGGAATCACAGGATTCAAGCGTTAGCAGATATTTCCCGTATTTTTGAGACTAAG*TGA*TAAAAAGATTGTGTCAGTTTTGTGACAGAGACCTAACAAACTATTTCAAGTAACCTAGTTTGTTACAAGGAATAGACAGTTCTGTTAAATAGGCCCGAGAGGGCTTTTTTTCTACATTTTTTGTGTTATCATAGACAGGTACTCATTTGAAAGGAATTTGAAAGAATGAAAATTTGTTTGATTTTTAATGGATAATGTGATATAATGGTTCAACAAACGAAAATTGGATAAAGTGGGATATTTTTAAAATATATATTTATGTTACAGTAATATTGACTTTTAAAAAAGGATTGATTCTAATGAAGAAAGCAGACAAGTAAGCCTCCTAAATTCACTTTAGATAAAAATTTAGGAGGCATATCAAATGAACTTTAATAAAATTGATTTAGACAATTGGAAGAGAAAAGAGATATTTAATCATTATTTGAACCAACAAACGACTTTTAGTATAACCACAGAAATTGATATTAGTGTTTTATACCGAAACATAAAACAAGAAGGATATAAATTTTACCCTGCATTTATTTTCTTAGTGACAAGGGTGATAAACTCAAATACAGCTTTTAGAACTGGTTACAATAGCGACGGAGAGTTAGGTTATTGGGATAAGTTAGAGCCACTTTATACAATTTTTGATGGTGTATCTAAAACATTCTCTGGTATTTGGACTCCTGTAAAGAATGACTTCAAAGAGTTTTATGATTTATACCTTTCTGATGTAGAGAAATATAATGGTTCGGGGAAATTGTTTCCCAAAACACCTATACCTGAAAATGCTTTTTCTCTTTCTATTATTCCATGGACTTCATTTACTGGGTTTAACTTAAATATCAATAATAATAGTAATTACCTTCTACCCATTATTACAGCAGGAAAATTCATTAATAAAGGTAATTCAATATATTTACCGCTATCTTTACAGGTACATCATTCTGTTTGTGATGGTTATCATGCAGGATTGTTTATGAACTCTATTCAGGAATTGTCAGATAGGCCTAATGACTGGCTTTTATAACAATGAATGGATAAACCCGACTTGGTAACATCATTTTGACGAATGAGATCTAGCTTTCGTGATAGGAAGCGATTCTCGTTCGTTTTTTCTTTGTCATACTCTTCGAAAATCTCTTCAAACCACGTCAGTTTTATCTGAAACCTCAAAGCTGTGCTTTGAGCAACCTGCGACTAGCTTTCTAGTTTACTCTTTGATTTTCATTGAGTATCAATTTGAATGGAAAATGGAAAGTTATTATCTTGTAATAATCCAAGCAACATTCTTGCAATCTATTTTACTTTATATCACAATTGATTGGTCAACTATTGATAAGTCAATGGATAGGAGGAAGAAATGATAGAGATTCAAGATTTACTGTATCAACTCCGCTTGTCTGAGCAAGCGAGTACGCAATTGTTTGAAAAAGGCTTGGGATTAGT*TTG*ACACGGTATCAGATTTTACTGTTTTTGCTGGAGCATTCTCCTTGTAACCAAATGGCGGTTCAGGAGCGTT
