# Supplementary material for: Protective Activity of Streptococcus pneumoniae Spr1875 Protein Fragments Identified Using a Phage Displayed Genomic Library
Source: PLoS One. 2012 May 3;7(5):e36588. doi: 10.1371/journal.pone.0036588 (PMC3343019; doi:10.1371/journal.pone.0036588)
Supplement: Text S1 — Supplemental Materials and Methods. (DOC) [file pone.0036588.s009.doc]

# Supplemental Materials and Methods

# Construction of pneumococcal mutans.

# The Δ*pspA* and Δ*spr1875* deletion mutants were constructed by gene SOEing, as described [21]. For the construction of the PspA-deficient strain, the *pspA* gene was substituted with an erythromycin-resistance cassette (*ermB*) using primers IF188/IF189 [22]. Oligonucleotides employed to amplify the regions upstream (822 bp) and downstream (565 bp) of the *pspA* gene are reported in supplemental Table S4. A 2571-bp long PCR fragment was used to transform D39, and an erythromycin-resistance mutant was selected and designated as Δ*pspA*. To generate the Spr1875-deficient strain, the *spr*1875 gene was replaced with a chloramphenicol-resistance cassette (*cat*) [23]. For this purpose, a 2007-bp DNA fragment containing the *cat* gene (850 bp) flanked by the regions upstream and downstream of *spr*1875 (665 bp and 492 bp, respectively) was generated and directly used to transform the D39 strain. The region upstream of *spr*1875 was amplified from the D39 chromosome using the primers listed in supplemental Table S4. The *cat* gene was amplified by using primers IF38/IF39. The *spr*1875-deficient strain was called Δ*spr1875*. Construction of both mutants was verified by PCR and sequencing.
